# Whole-Genome Analysis Revealed the Positively Selected Genes during the Differentiation of *indica* and Temperate *japonica* Rice

**DOI:** 10.1371/journal.pone.0119239

**Published:** 2015-03-16

**Authors:** Xinli Sun, Qi Jia, Yuchun Guo, Xiujuan Zheng, Kangjing Liang

**Affiliations:** 1 Key Laboratory of Ministry of Education for Genetics, Breeding and Multiple Utilization of Crops, Fujian Agriculture & Forestry University, Fuzhou, China; 2 College of Crop Science, Fujian Agriculture & Forestry University, Fuzhou, China; Institute of Crop Sciences, CHINA

## Abstract

To investigate the selective pressures acting on the protein-coding genes during the differentiation of *indica* and *japonica*, all of the possible orthologous genes between the Nipponbare and 93–11 genomes were identified and compared with each other. Among these genes, 8,530 pairs had identical sequences, and 27,384 pairs shared more than 90% sequence identity. Only 2,678 pairs of genes displaying a Ka/Ks ratio significantly greater than one were revealed, and most of these genes contained only nonsynonymous sites. The genes without synonymous site were further analyzed with the SNP data of 1529 *O*. *sativa* and *O*. *rufipogon* accessions, and 1068 genes were identified to be under positive selection during the differentiation of *indica* and temperate *japonica*. The positively selected genes (PSGs) are unevenly distributed on 12 chromosomes, and the proteins encoded by the PSGs are dominant with binding, transferase and hydrolase activities, and especially enriched in the plant responses to stimuli, biological regulations, and transport processes. Meanwhile, the most PSGs of the known function and/or expression were involved in the regulation of biotic/abiotic stresses. The evidence of pervasive positive selection suggested that many factors drove the differentiation of *indica* and *japonica*, which has already started in wild rice but is much lower than in cultivated rice. Lower differentiation and less PSGs revealed between the Or-It and Or-IIIt wild rice groups implied that artificial selection provides greater contribution on the differentiation than natural selection. In addition, the phylogenetic tree constructed with positively selected sites showed that the *japonica* varieties exhibited more diversity than *indica* on differentiation, and Or-III of *O*. *rufipogon* exhibited more than Or-I.

## Introduction

Asian cultivated rice (*O*. *sativa* L.) is one of the oldest and most important crop species. It is the primary source of food and livelihood for more than a third of Asia’s population, accounting for 35–60% Asia’s and ~20% the world’s caloric intake respectively[1]. *O*. *sativa* has a broad geographic distribution across the world with a high phenotypic variability, an estimated 120,000 varieties [1]. Most varieties have been placed into two subspecies, *O*. *sativa* ssp. *indica* and *O*. *sativa* ssp. *japonica*, which differ in more than 40 characteristics, such as phenol reaction phenotype, KClO_3_ resistance, cold sensitivity, drought tolerance, germination, seed shedding, length-width ratio of spikelet, apiculus hair length, awn length, digestion of endosperm in KOH solution, hardening of endosperm and first internode [2]. Some of these characteristics have been used to distinguish the *indica* and *japonica* varieties [2, 3]. Further analyses with ecological traits, isozymes and/or DNA markers confirmed and developed the above classification [2–8]. Studying 950 accessions with 4.1 million SNPs, Huang et al further divided the *japonica* subspecies into two sub-groups, temperate *japonica* and tropical *japonica*, and the *indica* subspecies into *indica* and aus sub-group [9].

The immediate progenitor of *O*. *sativa* is *O*. *rufipogon* [10]. Previous studies mostly focused on the domestication of wild rice, indicating that *O*. *sativa* was domesticated from *O*. *rufipogon* approximately 8200–13,500 years ago [2, 11]. However, these studies have provided two hypotheses about the origin(s) of two subspecies. One proposed that the domesticated rice originated from a single common wild ancestor, and differentiation of *indica* and *japonica* occurred after the domestication of cultivated species, which is supported by the analyses of well-characterized domestication genes and SNPs from 630 gene fragments in wild and cultivated rice accessions [11–16]. The other hypothesis suggested that two major rice types were domesticated separately from different populations of wild rice, supported by phylogenetic analyses that showed distinct clades in *O*. *sativa* for *indica* and *japonica* with different *O*. *rufipogon* accessions associated with each clade [17–22], as well as the whole-genome SNPs analyses [10, 23]. The SNPs analyses further indicated that *japonica* was first domesticated from a specific population of *O*. *rufipogon* around the middle area of the Pearl River in southern China, and that *indica* was subsequently developed from crosses between *japonica* and local wild rice as the initial cultivars spread into South East and South Asia[10].

Incomplete observations with one or several isozymes or domestication-related genes have only dropped small hints about the domestication processes, whereas the application and development of molecular markers can provide considerable information for the understanding of rice evolution. However, analyses with molecular markers such as RFLPs, SSRs and SNPs, which are usually caused by mutation, need to specify whether the mutation is neutral or not. A neutral mutation cannot change the gene function, and has no effect on fitness. Thus, such mutations provide less useful information for evolutionary analyses, and may interfere with the prediction. An advantageous mutation would have a positive effect on phenotype, and increase the fitness of the organism. These mutations will be accumulated in the gene pool. Conversely, deleterious mutations would decrease the fitness of the organism, and get typically eliminated from the gene pool by selection. Thus, positively selected genes (PSGs) carry much more information that is relevant to the evolutionary history of a species than negatively selected genes. Furthermore, the PSGs are, or have been, functionally important, and identification will facilitate the understanding of genetic variation that contributes to phenotypic diversity, and help to annotate the functional genome. Therefore, the emphasis of the domestication and differentiation analyses should be placed on the PSGs.

Differentiation of *indica* and *japonica* was driven by both artificial and natural selection, which directly acted on many characteristics [2, 24–27]. Selection results in a difference in gene frequencies between populations. Various factors have been known to be as selective forces, *e*.*g*., temperature, light condition, day length, soil fertility, stress conditions like drought, submergence, salinity, pollution, and herbicide use [2]. Protein evolution is the outcome of interaction between mutational processes and selective forces; therefore, analyzing the coding region of a genome is fundamental to understand how selection influences evolution. As a model organism, *O*. *sativa* is a well-characterised species with a small genome (389 Mb). The *indica* variety 93–11 and the *japonica* variety Nipponbare have been fully sequenced [28], and more than 2000 accessions including wild rice have been partly sequenced (http://ricevarmap.ncpgr.cn/django/home/) [10]. These features afford unique opportunities to explore differentiation of *indica* and *japonica* via genomic approaches. In this study, the genes under positive selection were identified and analyzed systematically in order to provide information for further understanding of cultivated rice evolution.

## Results

**Fig 1:**
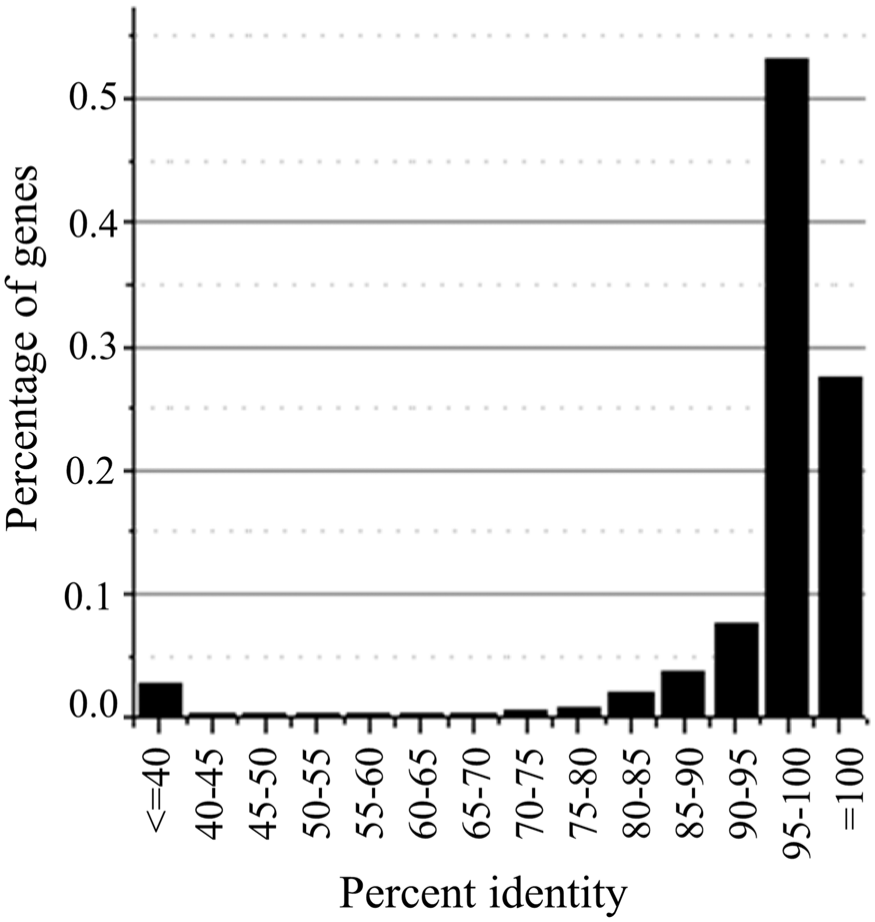
The distribution of the percent identity between the possible orthologs. The most similar proteins between 93–11 and Nipponbare were selected with BLAST, and 30995 pairs of proteins were obtained; each pair was analyzed via ClustalW 2 to obtain the percent identity.

All of the gene annotations for Nipponbare and 93–11 were downloaded from online public databases. There were 40,354 and 67,393 annotations for Nipponbare from the Rice Annotation Project Database (RAP-DB) and Rice Genome Annotation Project (RGAP), respectively, and 40,745 annotations for 93–11 from Rice Information System (RISe). The annotations of RGAP contained more alternatively spliced genes, transposons and retrotransposon genes. The databases of Nipponbare protein sequences retrieved from RAP-DB and RGAP were queried with the 93–11 protein sequences to identify pairs of orthologs, respectively. Combining the two BLAST results, 30,995 pairs of orthologous genes were found. The identity values of these orthologs were re-calculated according to the ClustalW2 result, and the resulting distribution of percent identities was shown in Fig. 1A total of 8,530 gene pairs exhibited 100% identity, and 27,384 pairs had more than 90% identity. These pairs were used to evaluate positive selection between *indica* and *japonica*.

### Positively selected genes between 93–11 and Nipponbare

**Fig 2:**
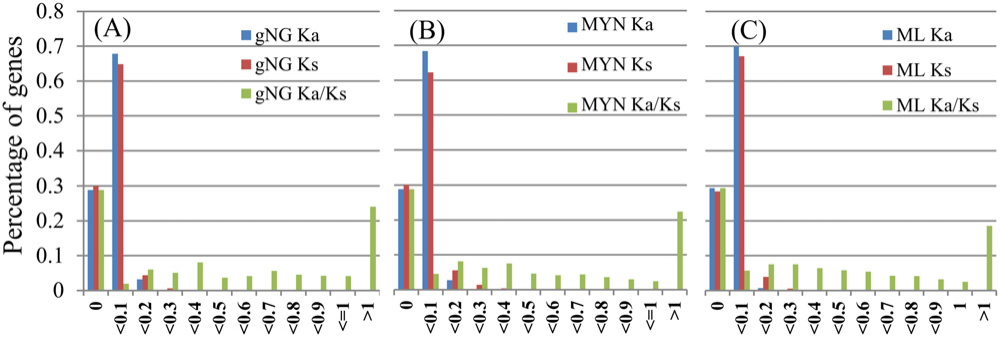
The distribution of Ka, Ks and Ka/Ks. (A) Using the gNG method. (B) Using the MYN method. (C) Using the Maximum Likelihood method. Note: Ka/Ks were specified as zero if both Ka and Ks were zero (5247 genes).

Positive selection is often evaluated by the ratio of nonsynonymous/synonymous substitution rates. This ratio, Ka/Ks, is expected to be greater than 1.0 in the case of positive selection [29]. The orthologous genes with high identity values (>90%) were used to detect instances of positive selection between 93–11 and Nipponbare by estimating their synonymous and nonsynonymous substitution rates. The Ka and Ks values of those genes were obtained with NG, gNG, YN, MYN, and maximum likelihood, respectively, and the results with maximum likelihood, gNG and MYN were shown in Fig. 2. The distributions of the Ka and Ks values were very narrow, with 99% of those genes displaying the Ka and Ks values below 0.3, and 18.5–24.5% of the gene pairs (more than 5000 pairs) showed Ka/Ks > 1 (Fig. 2). Fisher’s test was used to identify the Ka/Ks ratios that were significantly higher than one, suggesting more than 10% genes were positively selected during the differentiation of 93–11 and Nipponbare. In addition, the average percent identity of the PSGs was 99.29%.

**Fig 3:**
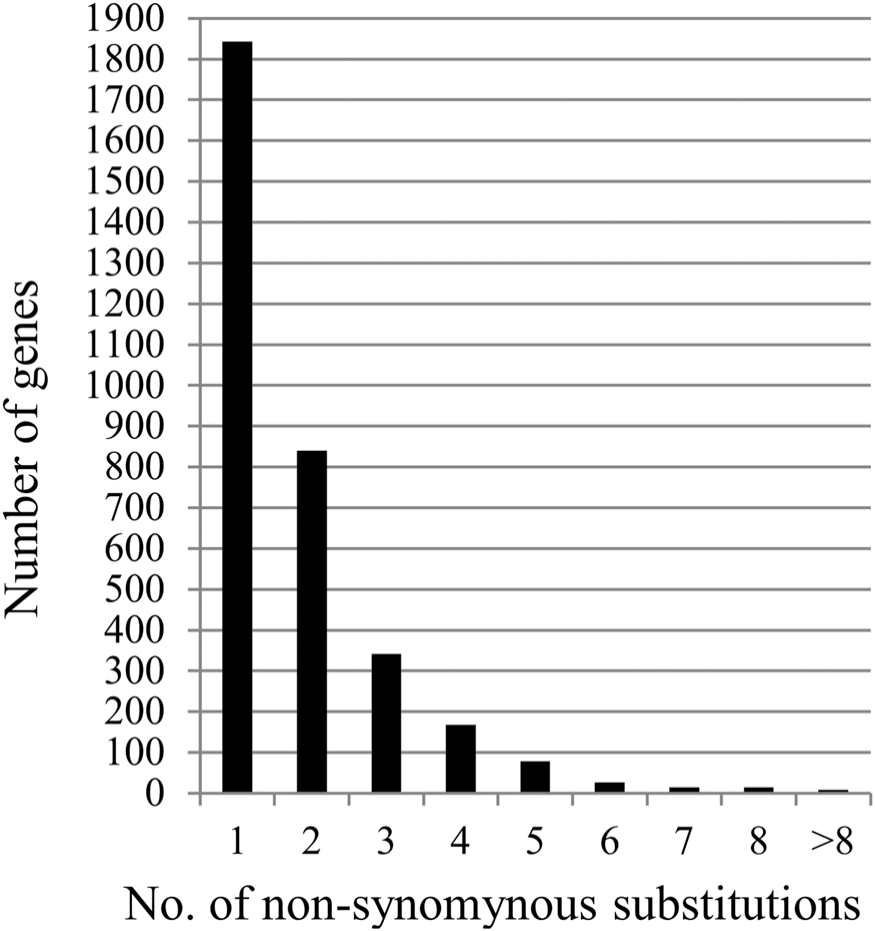
The distribution of the numbers of non-synonymous substitutions in NSSGs.

These PSGs were manually analyzed to remove annotation mistakes and ClustalW errors, followed by re-calculation. There were 2,977 PSGs detected with the gNG method and 2,799 PSGs with MYN. Among them, 2,664 PSGs were shared via both approaches. Interestingly, the synonymous substitution numbers of most these PSGs are zero (S1 Table). We denoted such type of genes as nonsynonymous substitution genes (NSSGs) including the genes whose Ka/Ks ratios were not significantly higher than one in Fisher’s test (S1 Table). Most of the NSSGs exhibited one or two substitutions (Fig. 3). Only seven PSGs with synonymous substitution sites were detected by at least one method (S2 Table).

To further investigate the selective pressure acting on protein-coding genes, all of the genes whose Ka/Ks ratios is not significantly higher than one were analyzed with an alternative approach to calculate the Ka/Ks ratios on sliding windows of fixed size. The alignment slicing procedure with sliding windows of 100 codons and a window shift of 34 codons generated 108,358 windows with less than 50% gap. Only 14 PSGs were identified by at least one of above methods (MYN, YN, NG and ML) after removing the annotation mistakes and ClustalW errors (S3 Table). Because it is difficult to do further analysis with the SNP data for these genes, we focused on NSSGs in the following study.

### SNP data can be used to estimate the positively selected sites in NSSGs between *indica* and temperate *japonica*

To detect whether the above PSGs found between 93–11 and Nipponbare were also under selection among most of the other *indica* and *japonica* accessions, the data containing 520 *indica* (excluding aus rice) and 409 temperate *japonica* accessions with 4.1 million SNPs was downloaded [10] and analyzed, as 93–11 belonged to *indica* and Nipponbare to temperate *japonica*. The SNPs in the exons of all PSGs were exposed, and most of the positively selected sites (PSSs) in the PSGs were able to be found in these SNPs. In addition, some new sites that changed amino acids were also discovered. Taking chromosome 1 for example, we found 2847 SNPs in the exons of 497 NSSGs, which included 687 (77.6%) PSSs revealed between 93–11 and Nipponbare. There were 198 (22.4%) PSSs that did not contain in these SNPs, so we speculated that these PSSs were specific to the differences between 93–11 and Nipponbare or the SNP coverage was not enough.

We calculated the Fst value of each SNP site based on its frequency in *indica* and temperate *japonica*, which was thought to be a measure of population differentiation due to genetic structure [30–32]. Most of these SNP sites possess lower Fst values, with 70.6% less than 0.25 and 60.7% less than 0.1. These sites could less affect the early differentiation between *indica* and *japonica*, and thus we did not examine whether their alterations changed the amino acids. The frequencies of these SNPs were calculated in total, indicating that the minor base frequencies of 1687 (83.9%) SNP sites were less than 0.1. It showed that these SNPs merely affected a small amount of rice accessions when they changed the amino acids, explaining why so many SNPs could not be found in PSSs.

We found 115 new nonsynonymous sites and 80 synonymous sites in the SNP data (Table 1). The difference of 93–11 and Nipponbare could represent about 73.2% diversity among all the *indica* and temperate *japonica* accessions when the Fst values are not less than 0.25. This suggested that it was possible to find synonymous sites in NSSGs in the study of the differentiation of *indica* and temperate *japonica*. It is difficult to calculate the number of the synonymous sites in the unsequenced region, but the probability could be estimated in the sequenced region, and the two regions are supposed to have an identical probability. The average probability is about 11.0%, but the probability is much less for the NSSGs with higher Fst values (Table 1). These results inferred that SNPs data could be used to estimate the positively selected sites in NSSGs between *indica* and temperate *japonica*.

**Table 1:** Number of nonsynonymous sites and new synonymous sites of chromosome 1 between *indica* and temperate *japonica*.

Fst	shared^1^	New Nonsyn.^2^	New syn.^3^	total	Probability^4^
> = 0.95–1	204	9	6	219	0.027
0.9–0.95	45	6	5	56	0.089
0.8–0.9	59	8	11	78	0.141
0.7–0.8	52	14	7	73	0.096
0.6–0.7	37	10	14	61	0.230
0.5–0.6	45	12	8	65	0.123
0.4–0.5	41	18	9	68	0.132
0.3–0.4	32	17	8	57	0.140
0.25–0.3	17	21	12	50	0.240
< 0.25	155				

^1^Number of nonsynonymous sites (PSSs) shared by sequence and SNP analyses.

^2^Number of new nonsynonymous sites found between *indica* and temperate *japonica*

^3^Number of new synonymous sites found between *indica* and temperate *japonica*

^4^The probability of synonymous site occurred in NSSGs.

### Positively selected genes between *indica* and temperate *japonica*

We discovered 313 genes that only contain nonsynonymous sites (Fst > = 0.25) in chromosome 1 (S4 Table). To detect whether these SNP loci were the signatures of adaptation during the genetic differentiation between populations, Lositan, an Fst related statistic method, was employed to identify the outliers that were positively influenced by selection, acting on either the locus itself or the closely linked locus. We identified 105 (19.9%) outliers within 99% confidence interval and additional 56 (10.5%) within 95% confidence interval. Almost all of the sites whose Fst values are more than 0.95 were outliers. However, the sites with the Fst value of one were not able to be detected as outliers because one is the biggest Fst value, and the program cannot distinguish which is bigger between the simulation and the sample Fst. These sites should be under selection during the differentiation of *indica* and *japonica*, and have already been completely fixed. We also detected some outliers with lower Fst values, which would infer the recent selection occurred (Table 2).

**Table 2:** The distribution of NSSGs and outliers along Fst values between *indica* and temperate *japonica*, and the distribution of outliers along Fst values between Or-It and Or-IIIt.

Fst values	No. of NSSGs between *indica* and temperate *japonica* ^1^		No. of outliers between *indica* and temperate *japonica*		No. of outliers between Or-It and Or-IIIt^2^
	Chr.1	Chr.2–12	Chr. 1	Chr. 2–12	
1	76	224	99	252	30
0.95-<1	68	310	93	429	66
0.9–0.95	19	162	34	240	64
0.8–0.9	34	68	13	85	22
0.7–0.8	34	4			
0.6–0.7	21	7	1	3	
0.5–0.6	27	11	9	18	2
0.4–0.5	25	22	5	44	8
0.25–0.4	9	24	6	56	28
Total	313	832	161	1127	220

^1^Only the PSS with highest Fst was considered for some PSGs with more than one PSS.

^2^The number of the PSSs used to analyze *O*. *rufipogon* is 1354, including some of the PSSs from the PSGs with a synonymous site.

We detected 173 (55.3%) genes (including the genes with the site whose Fst is one) with positively selected outliers and only nonsynonymous sites (S4 Table). Natural and/or artificial selection could directly act on these genes. Some genes with more than one nonsynonymous site were measured again after combining the SNPs to get the haplotypes of the genes, but no additional genes were found to be under positive selection (data not shown).

We found some NSSGs with synonymous sites between *indica* and temperate *japonica*, in which some synonymous sites were detected as outliers, and these may be hitchhiking sites (S5 Table). The genes with higher Fst values nonsynonymous sites and lower synonymous sites were further analyzed, and those with nonsynonymous outliers should be under positive selection (S5 Table).

All NSSGs on the other chromosomes were analyzed between *indica* and temperate *japonica* with Lositan. The outlier sites corresponding to the amino residues and their positions on the proteins were shown in S6 Table. Additional 832 genes (1005 genes in total including chromosome 1) containing only nonsynonymous outlier sites were revealed (Table 2 and S6), and 83.6% of these genes have at least one high-Fst-value site (Fst > 0.9).

**Fig 4:**
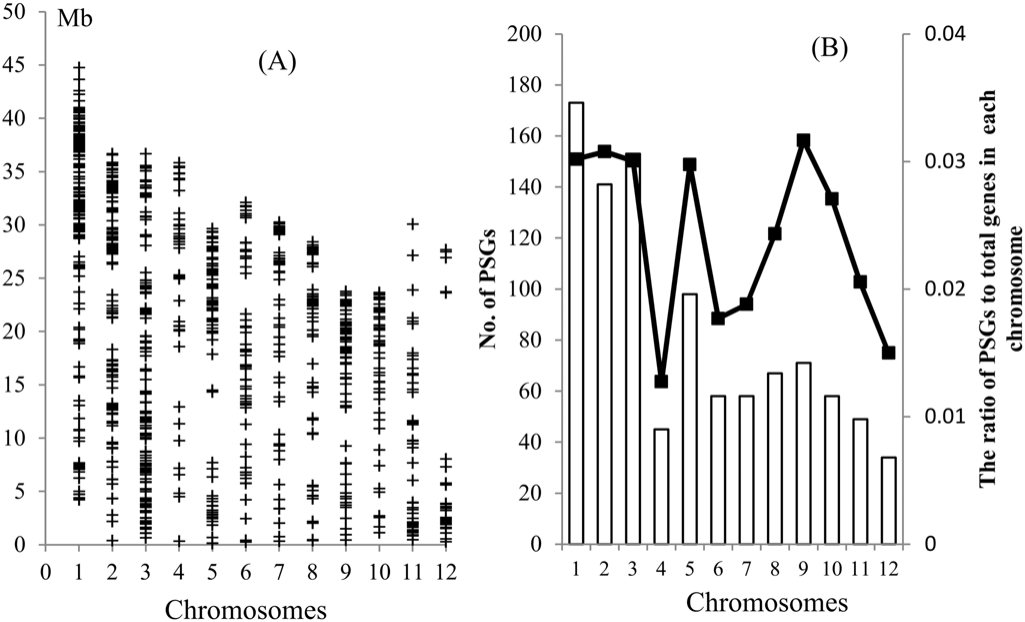
The distribution of the PSGs along rice chromosomes. (A) The distribution of the PSGs along the chromosomes. ‘+’ indicates the positions of the genes on the chromosomes. (B) The numbers of the PSGs (bars) and the ratios of PSGs (lines) to total genes in each chromosome.

The PSGs were unevenly distributed among and along the chromosomes (Fig. 4). The numbers of the PSGs in the chromosome 1, 2, 3 are about 2 times more than those in the chromosome 4, 6, 7, 10, 11 and 12 (Fig. 4B). The gene density was usually higher near the ends of the chromosomes, but the distribution patterns on different chromosomes were distinct. For example, the PSGs were preferentially located near the ends of the short arms of chromosome 11 and 12, and the ends of the long arms of chromosomes 1, 2, 4, 7, 8, 9 and 10 (Fig. 4A). The ratios of the PSGs to total genes in each million base pairs were calculated, and the pattern of these ratios along the chromosomes was similar but not identical to that of the numbers of the PSGs (S1 Fig). In addition, the distribution of the ratios of PSGs to total genes in each chromosome was similar with that of the numbers of the PSGs except chromosome 9, in which the gene density is much higher with respect to the number of PSGs (Fig. 4). The result indicated that the uneven distribution was independent on the gene density in a chromosome.

**Fig 5:**
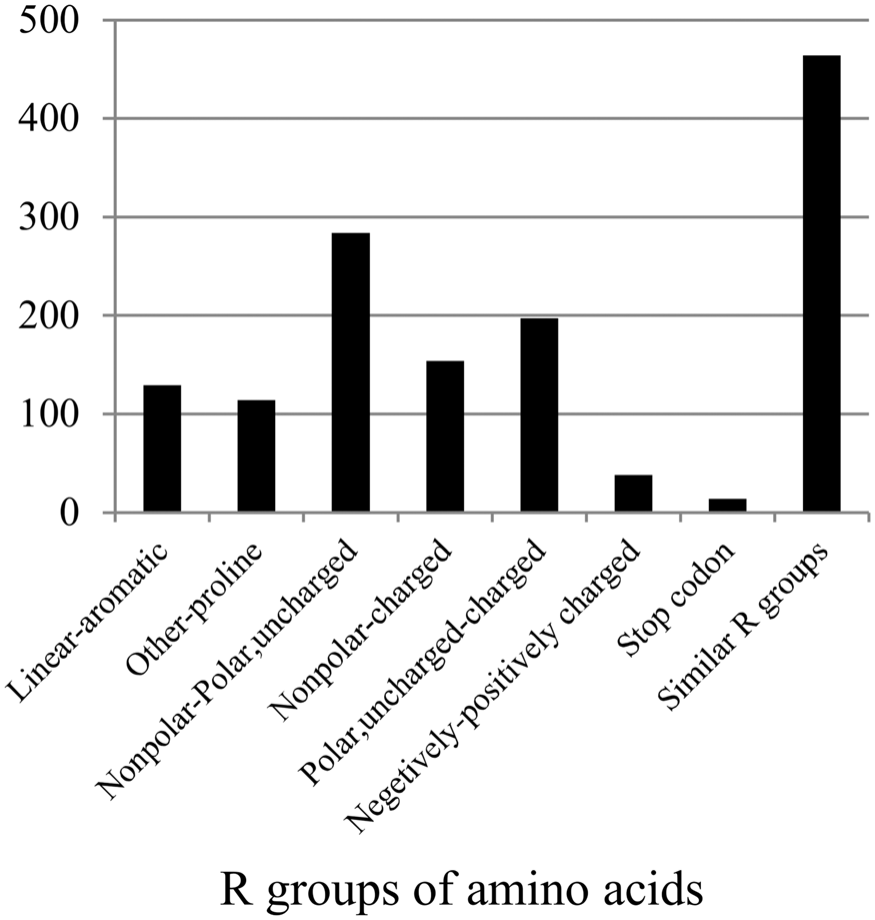
The number of each type of substitutions in the proteins encoded by the PSGs.

We found 1393 nonsynonymous outlier sites including one site whose change altered intron 3′ splice site and 14 sites whose changes resulted in stop codon. We found that 33.3% sites were replaced by the amino acids with similar R group (side chain), and the rest by the amino acids with different property (Fig. 5). Some substitutes were able to severely change the structures of the proteins, for example, the substitution of proline (Fig. 5).

We uncovered and selected additional 63 genes that contained only one synonymous site and some nonsynonymous outliers, in which the Fst value of the synonymous site was not higher than at least one nonsynonymous site (S7 Table). Some of these genes contained a nonsynonymous site, which altered the codon to stop codon, before the synonymous site on the gene. These genes were included in S7 Table as well, and could be under positive selection.

### Functional classification of PSGs

Based on the gene annotations, we summarized the possible functions of the PSGs (S8 Table). However, the function annotations of at least 223 PSGs are hypothetical or unknown, and some other PSGs contained two or more domains or motifs. Many proteins encoded by PSGs possessed zinc finger domain, binding domain, F-box domain or ankyrin repeat etc., or belonged to the protein families of transcription factor, transferase, protein kinase, peptidase, synthase, transporter or hydrolase etc. (S8 Table).

Blast2GO, RAP-DB and MSU Rice Genome Annotation Project were used to reveal the GOs of these PSGs, and cellular component, molecular function and biological process annotations were found in only 492, 626 and 499 PSGs, respectively. These genes were involved in 218 molecular functions and 213 biological processes, respectively (S9 Table and S10 Table). More attentions should be paid for 402 (64.2%) proteins (including sequence-specific DNA binding transcription factor activity) encoded by the PSGs with binding activity, 116 (18.5%) with hydrolase activity, 93 (14.9%) with transferase activity. There were 118 (23.6%) PSGs involved in macromolecular metabolic processes, 75 (15.0%) in biological regulation, 64 (12.8%) in transport (transmembrane transport and vesicle-mediated transport) and 63 (12.6%) in response to stimulus (including cellular response to stimulus). Some proteins could contain more than one activity (462 proteins) or be involved in more than one process (316 proteins), or vice versa.

To reveal whether the PSGs contained some genes of known function, we searched NCBI, QTARO (http://qtaro.abr.affrc.go.jp/ogro/table) and Google Scholar with the IDs of RAP-DB and RGAP. We found 29 genes have been characterized, and 47 genes’ expression patterns have been revealed (Table 3 and S11). More than half of these genes are involved in the regulation of biotic and/or abiotic stresses, and seven of these genes regulate the germ cell development (Table 3), especially *S5* gene (Os06t0213100 or LOC_Os06g11010), which regulates the hybrid sterility between *indica* and *japonica* variety [33] and was positively selected during differentiation of *indica* and *japonica* (S6 Table). The expressions of most genes can be induced or repressed by biotic and/or abiotic stresses (S11 Table). These results implied that many artificial/environmental factors could directly act on the genes and accelerated the variety differentiation. In particular, some known PSGs (*Dpl2* and *S5* genes) are involved in reproductive isolation (Table 3).

**Table 3:** The PSGs of known function.

Locus ID	Gene	Isolation or expression	Characters	Functions	Ref*
Os01t0678600	*asl1*	Mutant	Albino seedling lethality	Chloroplast development.	[34]
Os01t0695900	*OsMYB4*	Overexpression	Chilling and freezing tolerance	Cold tolerance	[35, 36]
Os01t0756700	*OsKAT1*	Overexpression	Salinity tolerance	Salinity tolerance in protoplast. Maintenance of cytosolic cation homeostasis.	[37]
Os01t0816100	*OsNAC4*	Knockdown	Blast resistance	Blast resistance. HR cell death.	[38–40]
Os01t0831000	*lax*	Mutant	Culm leaf, rachis-branches, lateral spikelet	Lateral organ development. Axillary meristem formation.	[41–44]
Os01t0867300	*Osabf1*	Mutant	Sensitive to drought and salinity treatment.	Drought and salinity tolerance.	[45]
Os01t0872800	*OsPdk1*	Mutant and overexpression	Overexpression of ospdk1 enhanced basal resistance against bacterial blight resistance and blast resistance	Ospdk1 participates in signal transduction through pathogen recognition	[46]
Os01t0929600	*rtS*	Knockdown	Sterility	Pollen development. Anther development.	[47]
Os02t0664000	*OsGPX3*	Knockdown	Dwarf and shorter root. Accumulation of H_2_O_2_	Root development. Dwarfism. H_2_O_2_ homeostasis.	[48]
Os02t0766700	*OsbZIP23*	Mutant and overexpression	Decreased sensitivity to ABA and tolerance to salinity and drought stress.	Drought and salinity tolerance. ABA sensitivity.	[49]
Os03t0119966	*rim1*	Mutant	Rice dwarf virus resistance.	Rice dwarf virus resistance.	[50]
Os03t0285800	*OsMAPK5 or OsMPK3*	Knockdown and Overexpression	Bacterial blight and blast resistance; cold, drought and salinity tolerance	Positively regulate response to biotic and abiotic stress, and JA pathway	[51–55]
Os03t0821300	*xb15*	Mutant	Bacterial blight resistance	Resistance to Xoo. Regulation of cell death.	[56]
Os05t0420300	*serf1*	Mutant	Sensitive to salt stress	Salinity tolerance.	[57]
Os06t0184100	*DPL2*	Natural variation	Sterility	Hybrid sterility. Pollen germination. Interaction with DPL1	[58]
Os06t0213100	*S5*	Natural variation	Sterility	Single locus hybrid sterility.	[33]
Os06t0354700	*nyc3*	Mutant	Stay green	Leaf senescence. Chlorophyll degradation.	[59]
Os06t0665400	*apo1 or SCM2*	Mutant and natural variation	Grain number. Lodging resistance	Floral organ identity; panicle branching; culm strength	[60–63]
Os06t0712700	*spw1 or OsMADS16*	Mutant and overexpression	Alter floral organ	Floral organ formation.	[64, 65]
Os06t0724900	*ila1*	Mutant	Increase leaf angle	Abnormal vascular bundle formation and cell wall composition in the leaf lamina joint.	[66]
Os07t0687700	*rTGA2*.*1*	Knockdown	Bacterial blight resistance and reduced plant stature	Resistance to Xanthomonas oryzae pv. Oryzae. Growth retardation.	[67]
Os08t0139000	*OsDEG10*	Knockdown	Sensitive to high light and cold stresses	High-light and cold tolerance.	[68]
Os08t0522400	*OsAPx-R*	Knockdown	Dwarf	Delay development and disturb steady state of the antioxidant	[69]
Os09t0439200	*OsJAZ8*	Overexpression	Bacterial blight resistance	JA induced resistance to Xanthomonas oryzae pv. Oryzae.	[70]
Os09t0441900	*DEP1*	Natural variation	Dense and erect panicle.	Enhance meristematic activity. Conferring cadmium tolerance	[71, 72]
Os09t0507200	*OsMADS8*	Knockdown	Panicle flower	Floral organ formation.	
Os09t0522000	*OsDREB1B*	Overexpression	Cold, drought and salinity tolerance	Regulators of the abiotic stress	[73–76]
Os09t0537700	*OsRNS4*	Overexpression	Salinity tolerance	Salinity tolerance. Positive regulation in ABA response	[77]
Os12t0572800	*mel2*	Mutant	Developmental aberration of germline and nursery cells	Regulate the premeiotic G1/S-phase transition of male and female germ cells,	[78]

*reference

### Differentiation and positive selection among the *O*. *rufipogon* accessions

*O*. *sativa* have been classified into five groups—*indica*, aus, temperate *japonica*, tropical *japonica* and intermediate, while *O rufipogon* into three groups—Or-I, Or-II and Or-III [9, 10]. To investigate the population differentiation on *indica* and *japonica*, we constructed a neighbour-joining tree with 446 *O*. *rufipogon* accessions and 1,083 *O*. *sativa* varieties based on the PSSs revealed between *indica* and temperate *japonica*, in which some PSSs were deleted for lack of data in some groups. The results showed that *indica* and temperate *japonica* were separately located on the two sides of the phylogenetic tree with the largest difference as prediction. The temperate and tropical *japonica* varieties distributed over a large range on the tree comparing with *indica* and aus which were clustered together. Most *indica* including the aus accessions seemed to generate from one progenitor. *O*. *rufipogon* were located between *indica* and *japonica*, meanwhile a small number of the Or-I accessions were in *indica* or aus group. Most of the intermediate varieties were located between tropical *japonica* and Or-III, Or-II and Or-III, or Or-I and Or-II. The aromatic varieties in the collection were put together with some intermediate varieties (S2A Fig). We also constructed a Minimum Evolution tree and obtained a similar result (data not shown). To explore the phylogenetic relationships of the wild rice further, we constructed a tree only with the *O*. *rufipogon* accessions. Most of Or-I and Or-II were concentrated together, whereas Or-III distributed over a large range. Unexpectedly, some of Or-III seemed more close to the Or-II group (S2B Fig). We selected some *O*. *rufipogon* accessions into three new groups—Or-It, Or-IIt and Or-IIIt for the next analysis, which were concentrated on the tree separately. Almost all of the accessions in Or-IIIt were from China.

The phylogenetic tree exhibited that the degree of the *indica-japonica* differentiation of the wild rice was between *indica* and *japonica*, and Or-IIt was between Or-It and Or-IIIt. We then measured the Fst values according to the frequencies of the PSSs, as shown in Table 4. The biggest average Fst value was between *indica* and temperate *japonica* as we expected, followed by the one between temperate *japonica* and Or-It. However, the Fst value between *indica* and Or-IIIt was much lower than that between temperate *japonica* and Or-It. The *indica-japonica* differentiation of the wild rice is mainly between Or-It and Or-IIIt, which exhibited a relatively high level of population differentiation (Table 4).

**Table 4:** The Fst values between the rice groups.

	Or-It	Or-IIt	Or-IIIt	TeJ*	TrJ*	*Indica*
Or-IIt	0.192					
Or-IIIt	0.476	0.247				
TeJ	0.802	0.561	0.230			
TrJ	0.658	0.452	0.198	0.076		
*Indica*	0.064	0.299	0.592	0.919	0.771	
aus	0.040	0.196	0.443	0.750	0.610	0.102

*TeJ: temperate *japonica*; TrJ: tropical *japonica*

The PSSs were also revealed to check whether they were outliers during population differentiation in the wild rice. It showed that 23.2% PSSs were found to be under positive selection between Or-It and Or-IIIt, but 29.7% outliers were associated with the Fst values lower than 0.25 (S12 Table). The GOs of these PSGs with the Fst values over 0.25 were shown in S12 Table, and no significant difference was found with the GOs’ distribution of *indica*~temperate *japonica*. The distribution of the outliers along Fst values were shown in Table 2. Most of the outliers with a high Fst value between Or-It and Or-IIIt also exhibited a high Fst value between *indica* and temperate *japonica* (S13 Table). These results inferred that the differentiation of *indica* and *japonica* should have started before domestication, and the differentiation was becoming stronger during and after domestication.

## Discussion

### The basis for understanding the differentiation of *indica* and *japonica*

The *indica* and *japonica* types exist as natural varieties that differ in their adaptation to distinct climatic, ecogeographic and cultural conditions [79]. The rice cultivars in the temperate countries such as Japan, Korea and northern China are exclusively *japonica*s, and those grown in the tropical and subtropical regions such as Thailand, Burma, India and southern China are usually *indica*s. In addition, some *japonica*s are also distributed in high altitude areas of the tropics [2]. Both natural and artificial selection have affected the distribution of *indica* and *japonica* rice, and brought about many different morphological and physiological traits between the two groups. Our study discovered that these PSGs were involved in 213 biological processes, and had 218 molecular functions except the genes without functional annotation. Many of these proteins encoded by the PSGs had binding activity, and were involved in response to stimulus and in biological regulation (S9 Table and S10 Table). More than half of the PSGs with known function and/or expression were involved in the responses to biotic/abiotic stresses, and some of them (*Dpl2* and *S5* genes) are involved in reproductive isolation (Table 3 and S11). These results implied that selection played an important role in the differentiation of *indica* and *japonica*, and these PSGs might directly or indirectly regulate and control these different traits. Further studies on the functions of these genes are essential to reveal the mechanism underlying the differentiation between varieties. Our results provided the basis for a comprehensive and systematic understanding of the differentiation of *indica* and *japonica*, and would help explain some important inter-subspecies differences. In addition, each target of positive selection has a story to tell about the historical forces and events that have shaped the history of a population.

### Whole-genome selection screening is necessary to study the differentiation of *indica* and *japonica*

By 4000 years ago, human societies worldwide had completed the domestication of all major crop species [80]. In the past ten or more years, researchers have identified the several specific genes that control some of the most important morphological changes associated with domestication. These genes include *tb1* [81] and *tga1* in maize [82]; *qSH1* [83], *sh4* [16], *Prog1* [12, 13] and *Rc* [84] in rice; *fw2*.*2* in tomato [85]; and the *Q* gene in wheat [86]. Although only a few domestication genes have been well documented, these analyses provided a great deal of information important to the understanding of how domestication modified plant development to produce today’s crops. Nevertheless, these data have not been sufficient to reveal the mechanism of domestication yet. Even fewer genes have been identified as involved in the differentiation of *indica* and *japonica*. Given this background, a whole-genome selection screen is a useful strategy for understanding the domestication and differentiation of *indica* and *japonica*. In this study, we revealed 1068 genes throughout the genome that underwent positive selection during differentiation, but found only 29 genes of known function. There were 15 genes involved in the regulation of biotic and/or abiotic responses; seven genes regulate the germ cell development. All of these genes except *S5* and *Dpl2* have not been reported to be involved in differentiation of *indica* and *japonica* in previous work. In addition, we found other 47 PSGs in response to various environment factors (Table 3 and S11). Our study laid the foundation for further research on evolution of cultivated rice.

### The large differences between the Nipponbare and 93–11 proteomes due to the differences of gene annotations

When the genes in Nipponbare and 93–11 were compared, orthologs could not be found for more than 10,000 genes. This suggested a major difference between the Nipponbare and 93–11 proteomes. However, for most of these genes, highly similar sequences were found in the Nipponbare or 93–11 genome when used as queries to search the other genome. This result implied orthologs could not be found for these genes mainly due to the differences in the annotations of Nipponbare and 93–11. Further evidence to support this view came from the two different systems used to annotate the Nipponbare genes, RAP-DB and RGAP. We used the 93–11 protein sequences as queries to search the RAP-DB and RGAP databases, and obtained 21,884 and 25,538 orthologs, respectively. When these two sets of results were combined, 30,995 pairs of orthologs were discovered. In addition, previous study showed that approximately one-third of the automated annotations contained errors in the NBS-LRR encoding genes in Arabidopsis, and more than one-third in LRR-kinase genes in rice [85, 87]. The results suggested that inadequate gene annotation was the main impediment to finding the orthologous relationships between the Nipponbare and 93–11 genes. In this study, we selected a lower standard to reveal all of the possible orthologs, and then found the PSGs with a higher standard and manually corrected the annotation and ClustalW mistakes. These greatly reduced error rate and workload.

### More genes than those detected were under positive selection during differentiation of *indica* and *japonica*

Several considerations make us to suppose that the actual number of the PSGs during differentiation of *indica* and *japonica* would be far more than that detected in this study. Firstly, some annotation errors brought about that no orthologs were found between 93–11 and Nipponbare. Secondly, some genes are pseudogenes in 93–11 or Nipponbare, but are functional genes in another. For example, the *phr1* gene lost its function due to an 18 nucleotide deletion in the *japonica* lines, but it remained functional in the *indica* lines [25]. The *Phr1* gene encoding a polyphenol oxidase controls the phenol reaction, which is an important trait for distinguishing *indica* and *japonica*. The grains of the *indica* cultivars turn brown after being soaked in phenol solution, whereas those of the *japonica* cultivars do not [2]. The genetic test revealed positive selection for the 18 bp deletion [25]. Unfortunately, our study failed to detect this selection on the *Phr1* gene because the method used in this research was not suited for analyzing deletion. Thirdly, the method based on the average Ka/Ks ratio over all the sites in a sequence is low powerful to detect positive selection comparing with PAML-codeml because adaptive evolution occurs at only a few sites, as most amino acids in a protein are under structural and functional constraints [88, 89]. That is why only a few PSGs with synonymous sites were revealed and so many NSSGs were discovered in this study. However, the recent data are not fit to PAML-codeml. Fourthly, we adopted a relatively stringent condition, which required that the PSGs should be detected by both of methods at the same time. It led to the results that some supposed PSGs would be excluded because of the stringent condition.

### Artificial selection accelerate *indica*-*japonica* differentiation

Our results indicated that the differentiation has already started in wild rice, but this differentiation is very low. Only 16.4% PSSs in *indica~japonica* were also detected in Or-It~ Or-IIIt, and the average Fst value between Or-It and Or-IIIt is 0.476 comparing with 0.919 between *indica* and temperate *japonica*. In addition, the populations of Or-It and Or-IIIt only constitute a small part of wild rice. If the differentiation in wild rice was supposed to be driven by natural selection, the *indica*-*japonica* differentiation in cultivated rice could be driven by natural and artificial selection. Moreover, artificial selection is much more powerful than natural selection on the differentiation.

### Differentiation of *indica* and *japonica* is one of the most important evolution directions

We used the PSSs to reconstruct the phylogenetic tree, which showed that temperate *japonica* is far from all of wild rice, but *indica* and Or-I were almost clustered together (S2A Fig). This result looks like that in the principal component analysis (PCA) plot of 1529 accessions with ~8 million SNP sites in the Huang’s paper (Supplementary Figure 13), in which the *japonica* varieties clearly segregate from the other groups, and some Or-I accessions mixed with the *indica* varieties [10]. This implied that *indica* came from Or-I, whereas *japonica* maybe derived from another wild rice group that is similar to Or-III and not included in the collection. However, Huang et al suggested that *japonica* was first domesticated from Or-III in southern China, and was subsequently crossed to Or-I wild rice in South East Asia and South Asia, thus generating *indica* after many cross-differentiation-selection cycles according to the analysis of domestication loci [10]. That seemed inconsistent with the results from all the SNP data [10] and our tree.

The first component in the PCA plot separated *indica* and *japonica*, and the second component separated *O*. *sativa* and *O*. *rufipogon* [10]. The result implied that differentiations of *indica* and *japonica*, and wild and cultivated rice are two main evolution directions. The differentiation of *indica* and *japonica* started in wild rice. The accessions of *japonica* and Or-III distributed over a large range in our tree, whereas that of *indica* and Or-I concentrated together. This inferred that the *japonica* varieties exhibited more diversity than *indica* on differentiation, Or-III than Or-I (S2 Fig). Thus, the study on the origin and evolution of *indica* and *japonica* should consider the power that acted on the differentiation. However, Huang’s model neglected the *indica-japonica* evolution direction [10], and thus could not explain the evolution of *indica* and *japonica* well.

## Materials and Methods

### The sequences and SNP data used in the study

The annotations of the genes of Nipponbare (temperate *japonica*) were downloaded from RAP-DB (http://rapdb.dna.affrc.go.jp/download/irgsp1.html) and Rice Genome Annotation Project (ftp://ftp.plantbiology.msu.edu/pub/data/Eukaryotic_Projects/o_sativa/annotation_dbs/pseudomolecules/version_6.1/all.dir/), respectively. The gene annotations for 93–11 (*indica*) were downloaded from the RISe database (ftp://ftp.genomics.org.cn/pub/ricedb/rice_update_data/GLEAN_genes/Beijing_indica/GLEAN_genes/). The genome DNA sequences of Nipponbare and 93–11 were obtained from the IRGSP/RAP build 5 dataset (http://rapdblegacy.dna.affrc.go.jp/download/index.html) and ricedb/RGPVs9311/9311 (ftp://ftp.genomics.org.cn/pub/ricedb/rice_update_data/genome/9311/), respectively. All of the SNP data were download from Rice Haplotype Map Project Database (http://www.ncgr.ac.cn/RiceHap2/index.html).

### Sequence alignment and the discovery of the orthologous genes between *indica* and *japonica*

The stand-alone BLAST programs (ncbi-blast-2.2.24+.exe; ftp://ftp.ncbi.nlm.nih.gov/blast/executables/blast+/LATEST/) were used to search each database with default parameters. The BLAST results were parsed with the Bio::SearchIO module in perl (http://search.cpan.org/~cjfields/BioPerl-1.6.901/Bio/SearchIO.pm). The results were manually treated with EXCEL to find the most similar genes on the collinear regions of the 93–11 and Nipponbare chromosomes, which were considered orthologous. The pairs of orthologs were aligned with ClustalW2 [90] using the Bio::Tools::Run::Alignment::Clustalw module (http://search.cpan.org/~cjfields/BioPerl-Run-1.006900/lib/Bio/Tools/Run/Alignment/Clustalw.pm). The percent identity values were calculated according to the ClustalW2 results. To convert a multiple sequence alignment of proteins to a codon alignment of the corresponding DNA sequences, PAL2NAL[91] was implemented using the Bio::Tools::Run::Alignment::Pal2Nal module (http://search.cpan.org/~cjfields/BioPerl-Run-1.006900/lib/Bio/Tools/Run/Alignment/Pal2Nal.pm).

### Analysis of selective pressure

Yang and Nielsen (YN) [92], maximum likelihood (ML) [93], Nei & Gojobori (NG) [94], MYN [95], gNG [96], and gMYN [96] methods were adopted to estimate the synonymous and nonsynonymous substitution rates in pairwise comparisons of protein-coding DNA sequences. The program PAML [97], Bio::Tools::Run::Phylo::PAML::Codeml (http://search.cpan.org/~cjfields/BioPerl-Run-1.006900/lib/Bio/Tools/Run/Phylo/PAML/Codeml.pm) and Bio::Tools::Run::Phylo::PAML::Yn00 modules (http://search.cpan.org/~cjfields/BioPerl-Run-1.006900/lib/Bio/Tools/Run/Phylo/PAML/Yn00.pm) were used to implement the YN, ML and NG methods; KaKs_calculator 2.0 [29] was used to execute the gNG, MYN and gMYN methods. The outcomes of these methods were compared, and only the results of gNG and MYN were further analyzed. The outputs were ordered by the Ka/Ks value and by the significance level of the associated Fisher’s test, which indicates whether the Ka/Ks ratio is significantly different from one.

The positions of the exons of the PSGs were revealed with Blast, and the SNPs in exons were selected, and their Fsts were calculated with EXCEL. All SNPs with more than 0.25 Fst values were manually checked to see if they changed the amino acids with the help of Sequencher (http://www.genecodes.com) and DNAsis Max Trial 1.0 (http://www.miraibio.com/download/).

Lositan, a Fst related statistic method, was employed to identify outliers for selection detection [98]. The phylogenetic tree was constructed with MEGA6 (http://megasoftware.net/).

### GO analysis

The GO annotations of the PSGs were retrieved from Rice Genome Annotation Project (http://rice.plantbiology.msu.edu/) and RAP-DB, or searched with Blast2GO using the default threshold [99, 100]. The GO annotations for biological processes, molecular functions and cellular component categories were classified into different groups with OBO-edit version 2.1.1 (http://sourceforge.net/projects/geneontology/files/). The chromosomal positions of the PSGs were recovered by searching the Nipponbare genome sequences, parsing the sequences with the Bio::SearchIO module and then mapping the resulting data with EXCEL.

## Supporting Information

S1 Table List of the genes whose Kss are zero and Kas are above zero. (XLSX)

S2 Table The positively selected genes detected with KaKs_calculator, whose Kss are more than zero. (XLSX)

S3 Table The positively selected genes detected with KaKs_calculator on sliding windows of fixed size. (XLSX)

S4 Table The NSSGs with outliers in chromosome 1. (XLSX)

S5 Table The NSSGs with outliers and synonymous sites between *indica* and temperate *japonica* in chromosome 1. (XLSX)

S6 Table The NSSGs with outliers in rice genome except chromosome 1. (XLSX)

S7 Table The genes that were selected as PSGs contained one synonymous site or nonsense mutant site. (XLSX)

S8 Table The possible functions of the PSGs. (DOCX)

S9 Table The molecular functions of the positively selected genes according to GO analysis. (XLSX)

S10 Table The biological processes of the positively selected genes according to GO analysis. (XLSX)

S11 Table The PSGs with the expression pattern found in the NCBI and Google scholar database. (DOCX)

S12 Table The comparison of the outliers revealed between Or-It and Or-IIIt, and between *indica* and temperate *japonica*. (XLSX)

S13 Table The Molecular functions and biological processes of the positively selected genes between Or-It and Or-IIIt according to GO analysis. (XLSX)

S1 Fig The ratios of PSGs to total genes in each Mb along the chromosomes. (TIF)

S2 Fig The population differentiation in 1529 rice accessions. (A) Neighbor-joining tree of 446 *O*. *rufipogon* accessions and 1,083 *O*. *sativa* varieties constructed with the PSSs. The five divergent groups, *indica*, aus, temperate *japonica*, tropical *japonica* and intermediate were indicated with different colors. The scale bar indicates the simple matching distance. (B) Neighbor-joining tree of 446 *O*. *rufipogon* accessions constructed with the PSSs.
(TIF)
