# Supplementary material for: Whole-Genome Analysis Revealed the Positively Selected Genes during the Differentiation of indica and Temperate japonica Rice
Source: PLoS One. 2015 Mar 16;10(3):e0119239. doi: 10.1371/journal.pone.0119239 (PMC4361536; doi:10.1371/journal.pone.0119239)
Supplement: S8 Table — (DOCX) [file pone.0119239.s008.docx]

| Table S8. The possible functions of the PSGs | |
| --- | --- |
| Function | No. the PSGs |
| Transcription factor | 43 |
| Zinc finger | 36 |
| Transferase | 35 |
| F-box | 34 |
| Binding domain, but no transcription factor | 33 |
| Protein kinase | 29 |
| Synthase | 20 |
| Peptidase | 19 |
| Pentatricopeptide repeat domain | 17 |
| Transporter | 17 |
| Hydrolase | 13 |
| Ribosomal protein | 12 |
| Alpha/beta hydrolase fold | 12 |
| transposon | 12 |
| protein phosphatase | 10 |
| Nuclease | 9 |
| Ankyrin | 9 |
| Phosphatase | 8 |
| Heat shock | 8 |
| dehydrogenase | 7 |
| Oxidoreductase | 7 |
| Kinase | 6 |
| Cytochrome P450 | 6 |
| Disease resistance response | 6 |
| MADS-box | 5 |
| Peroxidase | 5 |
| Pectinesterase inhibitor | 4 |
| Esterase | 4 |
| NB-ARC | 4 |
| Protease | 4 |
| WD40 | 4 |
| Auxin-responsive | 3 |
| Antiporter | 3 |
| Ubiquitin ligase | 3 |
| Proteinase | 2 |
