# Supplementary material for: Whole-Genome Analysis Revealed the Positively Selected Genes during the Differentiation of indica and Temperate japonica Rice
Source: PLoS One. 2015 Mar 16;10(3):e0119239. doi: 10.1371/journal.pone.0119239 (PMC4361536; doi:10.1371/journal.pone.0119239)
Supplement: S11 Table — (DOCX) [file pone.0119239.s011.docx]

Table S11. The PSGs with the expression pattern found in the NCBI and Google scholar database.

| RGAP-ID | RAP-DB-ID | cDNA | Description | Expression | reference |
| --- | --- | --- | --- | --- | --- |
| LOC_Os01g74140.1 | Os01t0972800-01 | AK110625 | WRKY1 (WRKY transcription factor 17). | Repressed by cold, heat and oxidative stress | [[1](#_ENREF_1)] |
| LOC_Os02g16730.1 | Os02t0267200-00 | CT837828 | Alpha-expansin OsEXPA13. | Repressed by Aluminum | [[2](#_ENREF_2)] |
| LOC_Os02g26430.1 | Os02t0462800-01 | AK110587 | WRKY transcription factor 42 (Transcription factor WRKY02). | Induced by cold, heat and oxidative stress | [[1](#_ENREF_1)] |
| LOC_Os01g64360.1 | Os01t0863300-01 | AK111571 | Similar to MCB2 protein. | Induced by brown planthopper resistance | [[3](#_ENREF_3)] |
| LOC_Os09g21180.1 | Os09t0379600-00 | CT837895 | Similar to Homeobox-leucine zipper protein HOX25. | Induced by brown planthopper resistance | [[3](#_ENREF_3)] |
| LOC_Os01g53040.1 | Os01t0730700-01 | AK109770 | WRKY transcription factor 14 (WRKY14). | Induced by brown planthopper resistance | [[3](#_ENREF_3)] |
| LOC_Os01g53220.1 | Os01t0733200-01 | AK066316 | Similar to Heat shock transcription factor 29 (Fragment). | Induced by brown planthopper resistance | [[3](#_ENREF_3)] |
| LOC_Os02g13520.1 | Os02t0228900-01 | AK121870 | Similar to Auxin-responsive protein IAA18 (Indoleacetic acid-induced protein 18). | Induced by brown planthopper resistance | [[3](#_ENREF_3)] |
| LOC_Os02g44930.1 | Os02t0670400-01 | AK242556 | Similar to HMGc1 protein. | Induced by brown planthopper resistance | [[3](#_ENREF_3)] |
| LOC_Os02g45670.1 | Os02t0680700-01 | AK288059 | Similar to DNA binding protein. | Induced by brown planthopper resistance | [[3](#_ENREF_3)] |
| LOC_Os05g44180.1 | Os05t0518000-00 | EU241924 | Similar to ZCN12 protein. | Involved in flower time and development, regulated by Ghd7 and Hd1 | [[4](#_ENREF_4)] |
| LOC_Os02g45570.2 | Os02t0678800-01 | AK108170 | Similar to Transcription activator. | Induced by brown planthopper | [[3](#_ENREF_3)] |
| LOC_Os02g54160.1 | Os02t0782700-02 | AK119885, AK064027 | Similar to Transcription factor EREBP1. | Regulate the expression of pathogenesis-related genes and drought and moderate cold response | [[5](#_ENREF_5),[6](#_ENREF_6)] |
| LOC_Os05g13830.1 | Os05t0225800-01 | AK070646 | Similar to Szp protein. | Repressed by Roc5 that modulate leaf rolling | [[7](#_ENREF_7)] |
| LOC_Os10g28350.1 | Os10t0419400-01 | AK102331, AK103834 | Similar to SIPL. | Involved in abiotic stress responses and tolerance | [[8](#_ENREF_8)] |
| LOC_Os04g46830.1 | Os04t0554800-01 | AK242066 | Similar to RCc3 protein. | Regulated by WRKY76 that plays dual and opposing roles in blast disease resistance and cold tolerance. | [[9](#_ENREF_9)] |
| LOC_Os10g27050.1 | Os10t0410600-02 | AK099604 | Similar to Phosphoprotein phosphatase 2A isoform 4. | Induced in all tissues at all development stages by drought stress | [[10](#_ENREF_10)] |
| LOC_Os10g40040.1 | Os10t0548100-01 | AK107224 | Similar to DM280 protein. | Induced in all tissues at all development stages by drought stress | [[10](#_ENREF_10)] |
| LOC_Os06g26270.1 | Os06t0367500-01 | AK063619 | Similar to B-cell receptor-associated protein 31-like containing protein. | Induced by drought in all leaves | [[10](#_ENREF_10)] |
| LOC_Os06g23350.1 | Os06t0341300-01 | AK107654 | Seed maturation protein domain containing protein. | Induced genes in all tissues at all development stages by drought | [[10](#_ENREF_10)] |
| LOC_Os03g20290.1 | Os03t0318400-01 | AK106440 | Peptidase A1 domain containing protein. | Induced by aluminum | [[2](#_ENREF_2)] |
| LOC_Os12g38290.1 | Os12t0571000-01 | AK241023 | Metallothionein-like protein type 1. | Involved in ROS homeostasis of anther | [[11](#_ENREF_11)] |
| LOC_Os07g37250.1 | Os07t0558500-01 | AK064914 | Inositol phosphatase-like protein. | Involved in the degradation of chlorophyll - protein complexes during leaf senescence | [[12](#_ENREF_12)] |
| LOC_Os09g24954.1 | Os09t0416500-01 | AK242007 | Hypothetical conserved gene. | The gene is regulated by osmgt1 that mediated alleviation of aluminum (Al) toxicity | [[13](#_ENREF_13)] |
| LOC_Os01g09700.1 | Os01t0192900-00 | BT068927 | 1-aminocyclopropane-1-carboxylate synthase family protein. | Induced by drought in leaves | [[10](#_ENREF_10)] |
| LOC_Os04g12970.1 | Os04t0206600-01 | AK106302 | UDP-glucuronosyl/UDP-glucosyltransferase family protein. | Suppressed by nitrogen satrvation | [[14](#_ENREF_14)] |
| LOC_Os08g43300.1 | Os08t0546400-01 | AK070009 | UBX domain containing protein. | Induced by heat stress | [[15](#_ENREF_15)] |
| LOC_Os05g45320.1 | Os05t0529400-01 | AK063846 | Similar to ubiquitin domain containing 1. | Induced by salt stress | [[16](#_ENREF_16)] |
| LOC_Os01g61830.1 | Os01t0834700-02 | AK058413 | Similar to nucleic acid binding protein. | Induced by heat stress | [[15](#_ENREF_15)] |
| LOC_Os02g54130.1 | Os02t0782300-01 | AK067047 | Similar to Heat shock-like protein. | Induced by heat stress | [[15](#_ENREF_15)] |
| LOC_Os03g12370.2 | Os03t0224700-01 | AK072571 | Similar to HSP protein (Fragment). | Induced by heat stress | [[15](#_ENREF_15)] |
| LOC_Os03g31300.1 | Os03t0426900-01 | AK069123 | Similar to Heat shock protein 101. | Induced by heat stress | [[15](#_ENREF_15)] |
| LOC_Os04g47620.1 | Os04t0563900-00 | NONE | Similar to H0409D10.5 protein. | Induced by nitrogen starvation | [[14](#_ENREF_14)] |
| LOC_Os05g24770.1 | Os05t0312500-01 | AK069307 | Reticulon family protein. | Induced by salt stress | [[16](#_ENREF_16)] |
| LOC_Os08g07690.1 | Os08t0173600-01 | AK062724 | Conserved hypothetical protein. | Up-regulated specifically in response to bacterial, parasite and fungal infections | [[17](#_ENREF_17)] |
| LOC_Os08g42400.1 | Os08t0535800-02 | AK068776, AK061543, AK104766 | No apical meristem (NAM) protein domain containing protein. | Induced by salt stress | [[16](#_ENREF_16)] |
| LOC_Os10g35720.1 | Os10t0500700-01 | AK059628, AK067982 | Similar to Monothiol glutaredoxin-S11. | Induced by heat stress | [[15](#_ENREF_15)] |
| LOC_Os10g39190.1 | Os10t0537100-00 | FP096459 | Hypothetical conserved gene. | Repressed by PEG | [[18](#_ENREF_18)] |
| LOC_Os05g38980.1 | Os05t0465800-01 | AK072353 | Similar to RbohAp108. | Induced by SA stress | [[19](#_ENREF_19)] |
| LOC_Os07g28040.1 | Os07t0464200-00 | EU961745 | Similar to O-methyltransferase ZRP4. | Repressed by PEG | [[20](#_ENREF_20)] |
| LOC_Os11g19840.1 | Os11t0303600-01 | AK061247 | Similar to Herbicide safener binding protein. | Induced by drought stress | [[20](#_ENREF_20)] |
| LOC_Os04g57860.1 | Os04t0674800-01 | AK119913 | Similar to CEL1=CELLULASE 1 (Fragment). | Repressed in the mutant, Docs1, which involved in the specification of outer cell layers in rice roots | [[21](#_ENREF_21)] |
| LOC_Os02g51790.1 | Os02t0754300-01 | AK068621, AK059012, AK099805 | Ribosomal protein L29 family protein. | Repressed by BTH | [[22](#_ENREF_22)] |
| LOC_Os01g31980.1 | Os01t0504500-02 | AK103784 | Multi antimicrobial extrusion protein MatE family protein. | Induced by cadmium stress | [[23](#_ENREF_23)] |
| LOC_Os07g26150.1 | Os07t0443500-00 | CT836143 | Molecular chaperone, heat shock protein, Hsp40, DnaJ domain containing protein. | Induced by low nitrogen stress | [[24](#_ENREF_24)] |
| LOC_Os02g56940.1 | Os02t0814400-01 | AK120394 | Cytochrome c, monohaem domain containing protein. | Repressed by BTH | [[22](#_ENREF_22)] |
| LOC_Os05g46270.1 | Os05t0540100-01 | AK060623, AK103819 | Flap endonuclease-1a (EC 3.-.-.-) (OsFEN-1a). | Involved in plant DNA replication and repair | [[25](#_ENREF_25)] |

1. Mittal D, Madhyastha DA, Grover A (2012) Genome-wide transcriptional profiles during temperature and oxidative stress reveal coordinated expression patterns and overlapping regulons in rice. PLoS One 7: e40899.

2. Tsutsui T, Yamaji N, Huang CF, Motoyama R, Nagamura Y, et al. (2012) Comparative genome-wide transcriptional analysis of Al-responsive genes reveals novel Al tolerance mechanisms in rice. PLoS One 7: e48197.

3. Wang Y, Guo H, Li H, Zhang H, Miao X (2012) Identification of transcription factors potential related to brown planthopper resistance in rice via microarray expression profiling. BMC Genomics 13: 687.

4. Wang J, Yu H, Weng X, Xie W, Xu C, et al. (2014) An expression quantitative trait loci-guided co-expression analysis for constructing regulatory network using a rice recombinant inbred line population. J Exp Bot 65: 1069-1079.

5. Cheong YH, Moon BC, Kim JK, Kim CY, Kim MC, et al. (2003) BWMK1, a rice mitogen-activated protein kinase, locates in the nucleus and mediates pathogenesis-related gene expression by activation of a transcription factor. Plant Physiol 132: 1961-1972.

6. Serra TS, Figueiredo DD, Cordeiro AM, Almeida DM, Lourenco T, et al. (2013) OsRMC, a negative regulator of salt stress response in rice, is regulated by two AP2/ERF transcription factors. Plant Mol Biol 82: 439-455.

7. Zou LP, Sun XH, Zhang ZG, Liu P, Wu JX, et al. (2011) Leaf rolling controlled by the homeodomain leucine zipper class IV gene Roc5 in rice. Plant Physiol 156: 1589-1602.

8. Todaka D, Nakashima K, Shinozaki K, Yamaguchi-Shinozaki K (2012) Toward understanding transcriptional regulatory networks in abiotic stress responses and tolerance in rice. Rice 5: 6.

9. Yokotani N, Sato Y, Tanabe S, Chujo T, Shimizu T, et al. (2013) WRKY76 is a rice transcriptional repressor playing opposite roles in blast disease resistance and cold stress tolerance. J Exp Bot 64: 5085-5097.

10. Wang D, Pan Y, Zhao X, Zhu L, Fu B, et al. (2011) Genome-wide temporal-spatial gene expression profiling of drought responsiveness in rice. BMC Genomics 12: 149.

11. Hu L, Liang W, Yin C, Cui X, Zong J, et al. (2011) Rice MADS3 regulates ROS homeostasis during late anther development. Plant Cell 23: 515-533.

12. Yamatani H, Sato Y, Masuda Y, Kato Y, Morita R, et al. (2013) NYC4, the rice ortholog of Arabidopsis THF1, is involved in the degradation of chlorophyll - protein complexes during leaf senescence. Plant J 74: 652-662.

13. Chen ZC, Yamaji N, Motoyama R, Nagamura Y, Ma JF (2012) Up-regulation of a magnesium transporter gene OsMGT1 is required for conferring aluminum tolerance in rice. Plant Physiol 159: 1624-1633.

14. Cai H, Lu Y, Xie W, Zhu T, Lian X (2012) Transcriptome response to nitrogen starvation in rice. J Biosci 37: 731-747.

15. Jung K-H, An G (2012) Application of MapMan and RiceNet drives systematic analyses of the early heat stress transcriptome in rice seedlings. Journal of Plant Biology 55: 436-449.

16. Pandit A, Rai V, Sharma T, Sharma P, K. SN (2011) Differentially expressed genes in sensitive and tolerant rice varieties in response to salt-stress. Journal of Plant Biochemistry and Biotechnology 20: 149-154.

17. Narsai R, Wang C, Chen J, Wu J, Shou H, et al. (2013) Antagonistic, overlapping and distinct responses to biotic stress in rice (Oryza sativa) and interactions with abiotic stress. BMC Genomics 14: 93.

18. MA T-C, CHEN R-J, YU R-R, ZENG H-L, ZHANG D-P (2009) Global Genome Expression Analysis of Transcription Factors under PEG Osmotic Stress in Rice Root System. ACTA AGRONOMICA SINICA 35: 1030-1037.

19. Li1 Y, Chen Y, Wu J, He C (2011) Expression and functional analysis of OsRboh gene family in rice immune response. Chin J Biotech 27: 1574-1585.

20. Zhang XL, Liu M, Qi S, L. GC (2011) Response of active methyl cycle and transfer genes to drought stress in rice leaves. Chin J Rice Sci 25: 236-242.

21. Huang CF, Yamaji N, Ono K, Ma JF (2012) A leucine-rich repeat receptor-like kinase gene is involved in the specification of outer cell layers in rice roots. Plant J 69: 565-576.

22. Sugano S, Jiang CJ, Miyazawa S, Masumoto C, Yazawa K, et al. (2010) Role of OsNPR1 in rice defense program as revealed by genome-wide expression analysis. Plant Mol Biol 74: 549-562.

23. Ogawa I, Nakanishi H, Mori S, Nishizawa NK (2009) Time course analysis of gene regulation under cadmium stress in rice. Plant Soil 325: 97-108.

24. ZHAO M, MA D, WANG J, XU H, TANG L, et al. (2012) Expression of transcription factors of rice flag leaf under low nitrogen stress. Chin J Rice Sci 26: 275-282.

25. Kimura S, Furukawa T, Kasai N, Mori Y, Kitamoto HK, et al. (2003) Functional characterization of two flap endonuclease-1 homologues in rice. Gene 314: 63-71.
