# Supplementary figures and images for: Whole-Genome Analysis Revealed the Positively Selected Genes during the Differentiation of indica and Temperate japonica Rice
Source: PLoS One. 2015 Mar 16;10(3):e0119239. doi: 10.1371/journal.pone.0119239 (PMC4361536; doi:10.1371/journal.pone.0119239)

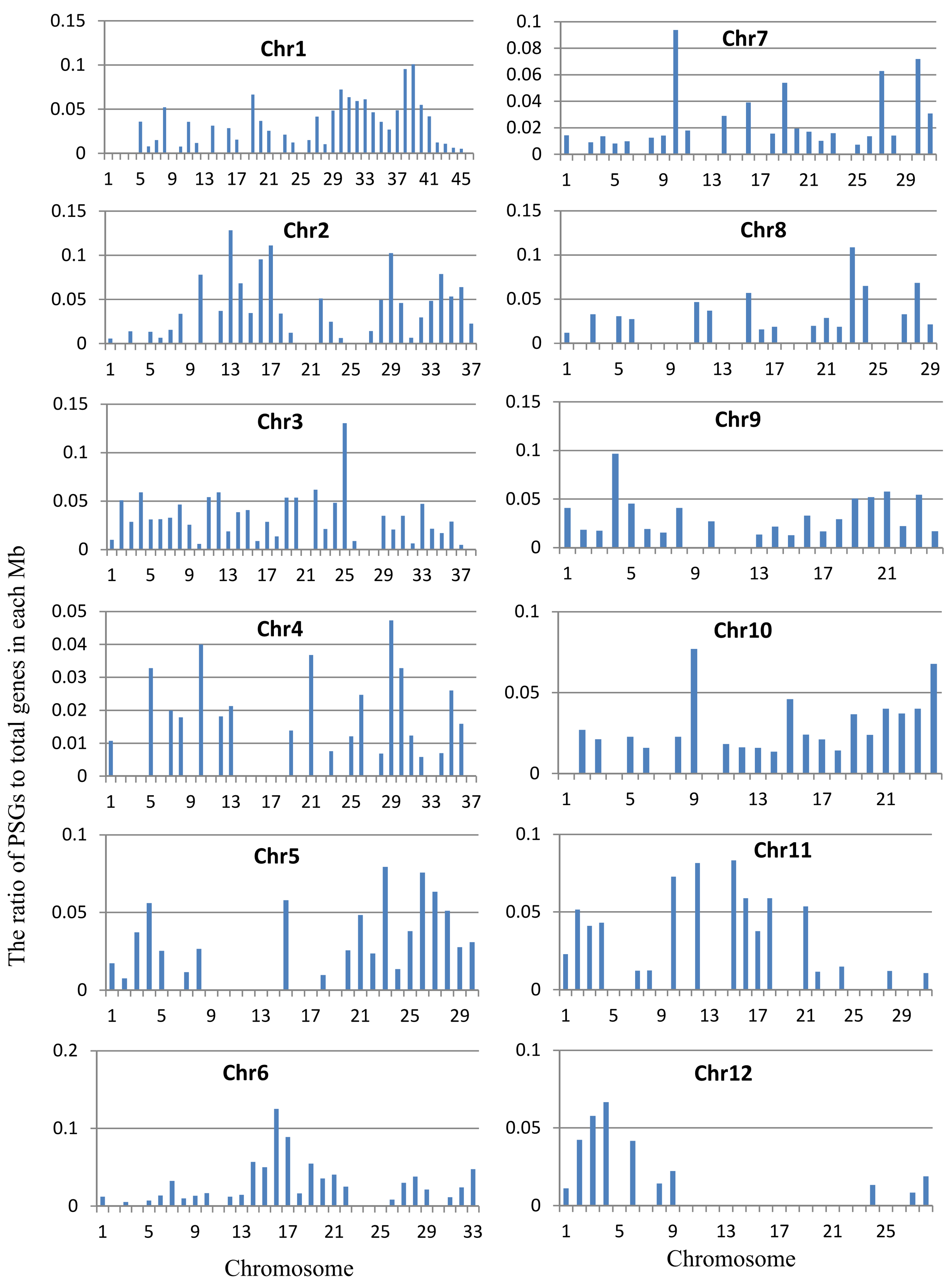

Supplement: S1 Fig — (TIF) [file pone.0119239.s014.tif]

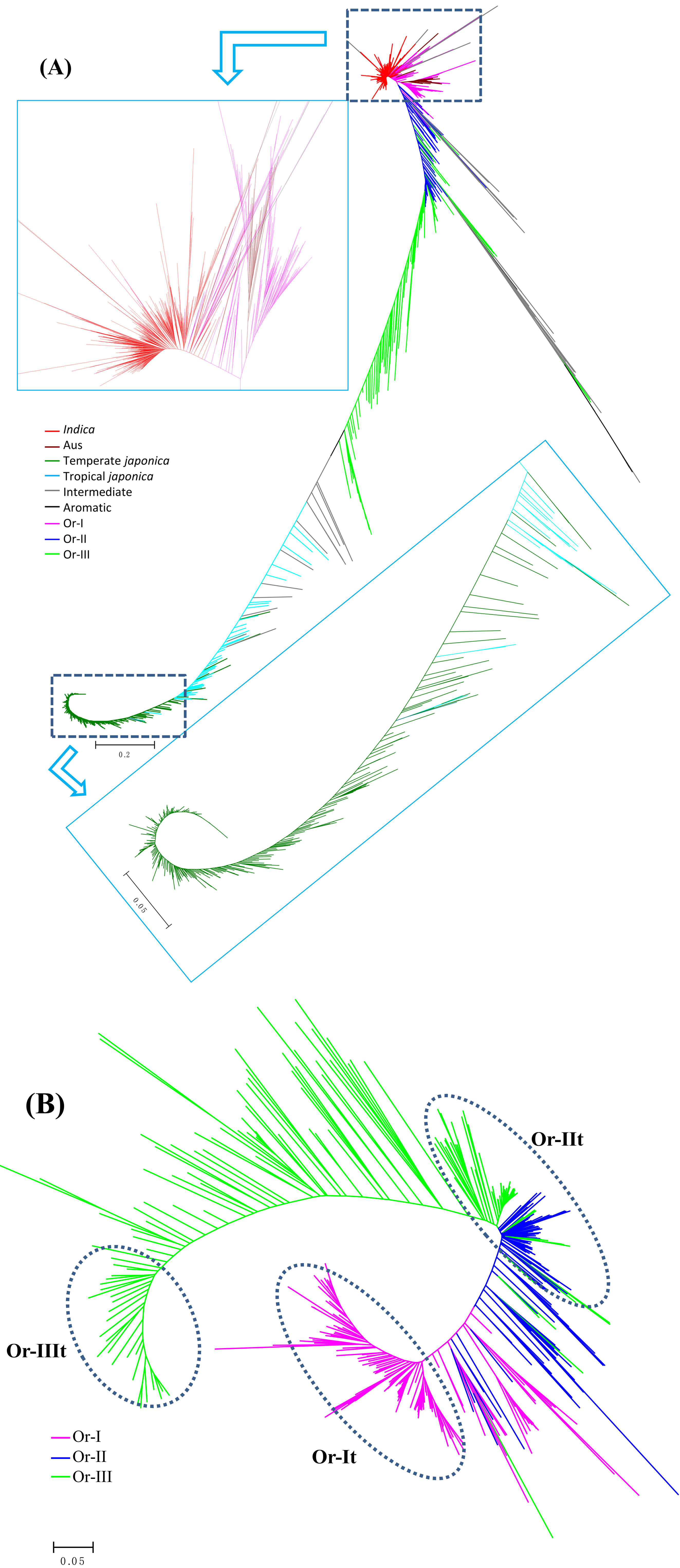

Supplement: S2 Fig — (A) Neighbor-joining tree of 446 O. rufipogon accessions and 1,083 O. sativa varieties constructed with the PSSs. The five divergent groups, indica, aus, temperate japonica, tropical japonica and intermediate were indicated with different colors. The scale bar indicates the simple matching distance. (B) Neighbor-joining tree of 446 O. rufipogon accessions constructed with the PSSs. (TIF) [file pone.0119239.s015.tif]
